# AtMYB72 as a Biotechnological Tool to Overcome Phenylpropanoid Substrate Limitation and Enhance Coumarin Biosynthesis in Plants

**DOI:** 10.1111/pbi.70503

**Published:** 2026-01-16

**Authors:** Jakob Weber Böhlen, Alexander Beesley, Sebastian F. Beyer, Patrick Schwinges, Alina E. Maas, Holger Schultheiss, Uwe Conrath, Caspar J. G. Langenbach

**Affiliations:** ^1^ Department of Plant Physiology RWTH Aachen University Aachen Germany; ^2^ BASF Plant Science Company GmbH Agricultural Center Limburgerhof Germany

**Keywords:** coumarin, metabolic engineering, MYB transcription factors, phenylpropanoid metabolism, plant, scopoletin, trait development, transgene

1

Plant secondary metabolites (PSM) provide mankind with nutrition, medicine and flavours. In plants, PSM fulfil major roles in stress tolerance, defence and interactions with beneficial or pathogenic organisms (Beyer et al. 2019; Selma et al. 2023; Stringlis et al. 2019). Many important classes of PSM—such as coumarins, flavonoids, monolignols, stilbenes, terpenoids, and diverse phenolic acids—originate from the phenylpropanoid pathway (PPP; Liu et al. 2015). Plant metabolic engineering (PME) enables targeted manipulation of biosynthetic routes to enhance the accumulation of metabolites that are beneficial to human or animal health, or that contribute to plant fitness (Selma et al. 2023). Several PME strategies have been successfully implemented in crops, with some traits reaching commercial application (Selma et al. 2023). One prominent example is the co‐expression of *AtMYB12* with other structural genes for enhancing the production of flavonoids in tomato (Zhang et al. 2015). MYB transcription factors (TFs), particularly those of the R2R3‐MYB family, are key regulators of primary and secondary metabolic pathways, including the PPP (Liu et al. 2015).

In this study, we aimed at utilising AtMBY72—a MYB‐family TF that is described to enhance transcription of biosynthetic genes in the shikimate pathway and early steps of the PPP (Zamioudis et al. 2014)—to overcome phenylpropanoid precursor limitation and enhance accumulation of coumarins in plants. We do this at the example of scopoletin biosynthesis. Scopoletin is a well‐studied coumarin which provides an arsenal of beneficial properties, such as antioxidant, antifungal, antibacterial, antiviral, and insect deterrent activity (Beesley et al. 2023; Beyer et al. 2019; Stringlis et al. 2019). We previously demonstrated that scopoletin inhibits *Phakopsora pachyrhizi* spore germination in a dose‐dependent manner (Beyer et al. 2019). Moreover, stable overexpression of the Arabidopsis gene *feruloyl‐CoA 6′‐hydroxylase1* (*F6′H1*)—a key enzyme in scopoletin biosynthesis—leads to constitutive scopoletin accumulation in transgenic Arabidopsis and soybean plants or tobacco BY2‐cells, resulting in attenuated mycotoxin‐induced oxidative stress and reduced disease susceptibility (Beesley et al. 2023). Likewise, overexpressing a cotton *F6′H1* (*GhF6′H1*) in cotton and a sweet potato *F6′H* (*IbF6′H2*) in tobacco enhanced resistance towards *Verticillium dahliae* and *Fusarium oxysporum*, respectively (Gao et al. 2024; Wang et al. 2024). Controlled elevation of the scopoletin content can therefore increase the agronomic, medicinal and nutritional value of crops.

Here, we demonstrate that AtMYB72 is a powerful tool for boosting production of the coumarin scopoletin and its glycosidic storage form scopolin by overcoming phenylpropanoid precursor limitation.

To assess whether scopoletin biosynthesis is limited by phenylpropanoid precursor availability, we fed ferulate to *AtF6′H1*‐overexpressing tobacco BY‐2 cells. Scopoletin levels increased 8‐fold within six hours post treatment (hpt) compared to the control (Figure 1b). These results indicate that ferulate is taken up by the BY2‐cells and converted to feruloyl‐CoA, presumably by endogenous 4‐coumarate‐CoA ligases (4CL; Figure 1a). Feruloyl‐CoA is the direct substrate of F6'H1 that catalyzes the formation of 6‐hydroxy‐feruloyl‐CoA—an intermediate in the formation of scopoletin (Figure 1a; Beesley et al. 2023; Stringlis et al. 2019). Over time, scopoletin levels declined while the abundance of the scopoletin‐glycoside scopolin gradually increased until 72 hpt (Figure 1b), implicating that scopoletin is glycosylated to scopolin (Figure 1a). This reveals that the biosynthesis of scopoletin and scopolin in *F6'H1*‐overexpressing cells is limited by phenylpropanoid precursor availability and *AtF6'H1*‐expression alone is not sufficient for the maximum biosynthesis of scopoletin and scopolin.

**FIGURE 1:**
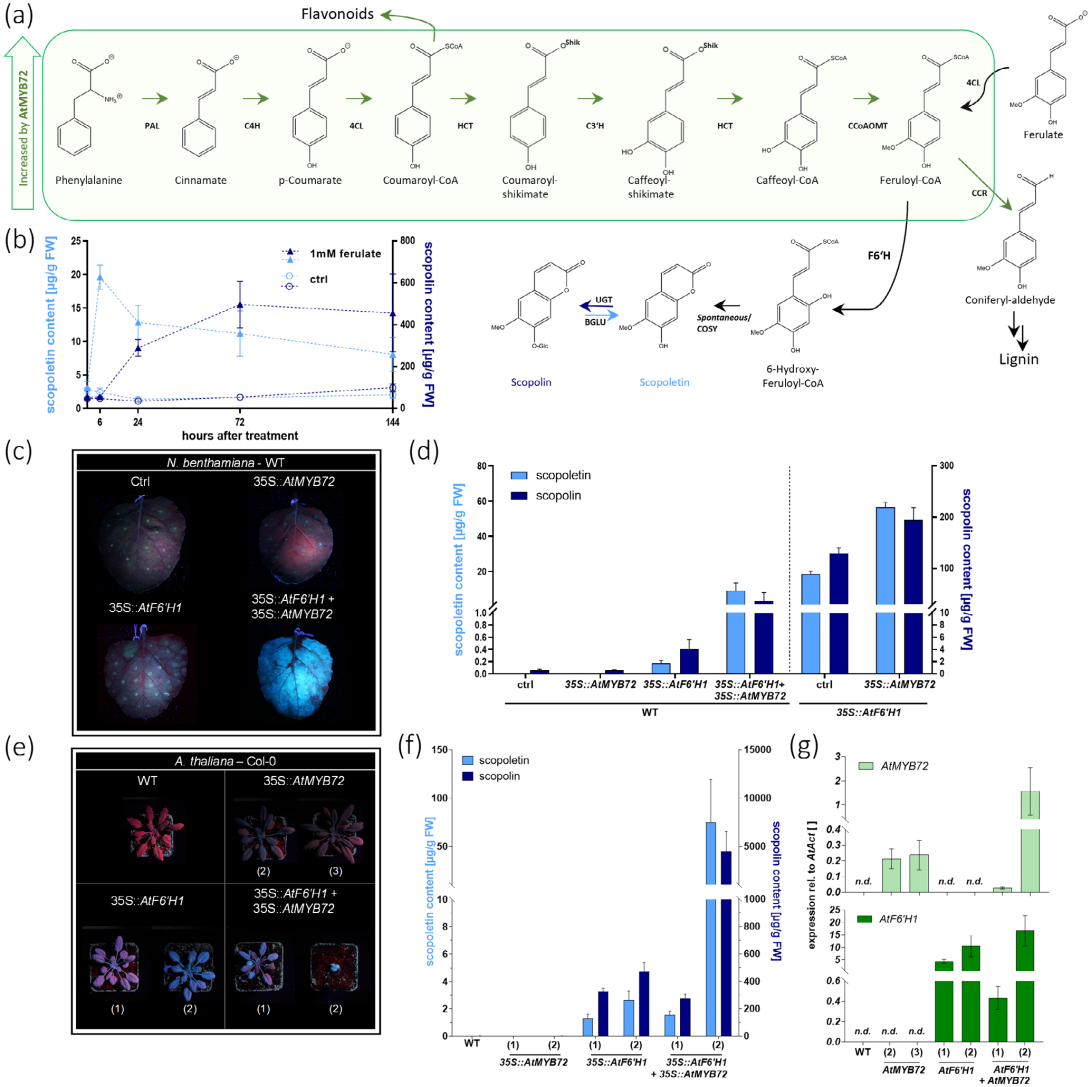
AtMBY72 overexpression increases scopoletin and scopolin content *in planta*. (a) Simplified scheme of scopoletin biosynthesis. (b) Transgenic (*F6ʹ‐H1‐*OE) BY2‐tobacco suspension +1 mM ferulate or 0.2% EtOH (control)—cell extract. (c and d) Transiently transformed *N. benthamiana* (WT and *F6′H1‐*OE)—UV images and leaf extracts. (e–g) Arabidopsis WT and transgenic (*MYB72*‐OE, *F6′H1*‐OE and *MYB72/F6H′1*‐OE) lines—UV images, leaf extracts and transgene expression. (Detailed description provided in Appendix S1.)

We overcame this bottleneck by transiently co‐overexpressing *AtMYB72* and *AtF6′H1* in *Nicotiana benthamiana*. This co‐overexpression resulted in a ~45‐fold increase in scopoletin accumulation in infiltrated leaves compared to those expressing *AtF6′H1* alone (Figure 1d). This vast increase in fluorescent scopoletin in transiently transformed leaves was also apparent in UV light. Leaves overexpressing *AtMYB72* and *AtF6′H1* fluoresce bright blue, while leaves overexpressing only one transgene showed none (*AtMYB72*) or weak blue fluorescence (*AtF6′H1*; Figure 1c). Scopoletin accumulation in *N. benthamiana* leaves transiently co‐expressing *AtF6′H1* and *AtMYB72* even reached similar levels as detected in leaves of *N. benthamiana* plants stably overexpressing *AtF6′H1* (Figure 1d). Transient co‐overexpression of *AtMYB72* in stable transgenic *AtF6′H1*‐overexpressing *N. benthamiana* plants boosted scopoletin (~50–60 μg/g FW) and scopolin (~200 μg/g FW) levels ~3 and ~2‐fold, respectively (Figure 1d). These findings demonstrate that *AtMYB72* effectively enhances phenylpropanoid precursor availability, thereby maximising coumarin biosynthesis. Moreover, *AtMYB72* can be deployed as an experimental tool, facilitating the detection of low‐abundant phenylpropanoid products in *Nicotiana benthamiana* transient expression assays.

In Arabidopsis plants stably co‐overexpressing *AtMYB72* and *AtF6′H1 (MYB72/F6H'1*‐OE‐lines*)*, we detected scopoletin and scopolin levels of ~75 μg/g FW and 5 mg/g FW, respectively (line #2; Figure 1f)—representing ~25—and 10‐fold increases compared to lines overexpressing *AtF6′H1* only (*F6′H1*‐OE‐line; Figure 1f; Beesley et al. 2023). However, strong transgene expression and scopoletin hyperaccumulation in this line were accompanied by a dwarf phenotype (Figure 1e), which is likely caused by redox misbalance in cells accumulating extremely high levels of the antioxidant scopoletin (Beesley et al. 2023; Beyer et al. 2019). In this case, scopoletin production exceeds the plant's endogenous capacity to glycosylate scopoletin to scopolin and store the inactive glycoside in the vacuole. Notably—in line with Zamioudis et al. (2014)—overexpression of *AtMYB72* alone led to increased expression of upstream genes in the PPP (Figure 1a, expression data in Figure S1). However, it neither provoked scopoletin accumulation nor led to any growth impairments (Figure 1d/f). This indicates that accumulation of scopoletin—and possibly, but less likely, scopolin—but not its upstream precursors are responsible for the stunted growth of *MYB72/F6′H1*‐OE‐line #2.

In contrast, *MYB72/F6'H1*‐OE line #1 exhibited approximately 10‐fold lower expression of both *AtMYB72* and *AtF6'H1* compared to the respective single‐gene overexpression lines (Figure 1g). Despite this, *MYB72/F6'H1*‐OE line #1 accumulated scopoletin and scopolin to levels similar to the *AtF6'H1*‐overexpressing controls (Figure 1f). This indicates that moderate stable co‐expression of the transcription factor *AtMYB72* and the biosynthetic gene *AtF6'H1* can yield a similar metabolic output as strong overexpression of *AtF6'H1* alone. Consequently, phenylpropanoid metabolism could potentially be engineered through minor combinatory modulations of endogenous PPP biosynthesis and MYB gene promoters rather than introducing foreign DNA and employing strong viral promoters. Such targeted promoter fine‐tuning could be achieved via site‐directed nuclease‐1 (SDN‐1)–mediated gene editing (Wang et al. 2025), TILLING, or EcoTILLING. Avoiding the introduction of foreign transgenes may shorten time‐to‐market and reduce regulatory costs for developing crop varieties with enhanced phenylpropanoid metabolite content. Identifying such GM‐independent strategies is of high practical relevance, given the regulatory and economic challenges associated with deploying GM crops in agriculture.

## Author Contributions

J.W.B., A.B., S.F.B., P.S., C.J.G.L., U.C. and H.S. designed the experiments. J.W.B., A.B., S.F.B., A.E.M. and P.S. performed the experiments. J.W.B., C.J.G.L. and U.C. wrote the manuscript. All authors revised the manuscript.

## Conflicts of Interest

The authors H.S., S.F.B., U.C., P.S. and C.J.G.L. are inventors of project‐linked patent WO2020120753A1. H.S. and S.F.B. are employees of BASF SE, A.B. is an employee of Betaseed, U.C., P.S. and C.J.G.L. are employees of AgPrime GmbH.

## Supporting information


**Appendix S1:** pbi70503‐sup‐0001‐Supinfo.doc.

## Data Availability

The data that support the findings of this study are available on request from the corresponding author. The data are not publicly available due to privacy or ethical restrictions.
