# Supplementary material for: AtMYB72 as a Biotechnological Tool to Overcome Phenylpropanoid Substrate Limitation and Enhance Coumarin Biosynthesis in Plants
Source: Plant Biotechnol J. 2026 Jan 16;24(5):3009–11. doi: 10.1111/pbi.70503 (PMC13110148; doi:10.1111/pbi.70503)
Supplement: Supplementary file 1 — Appendix S1: pbi70503‐sup‐0001‐Supinfo.doc. [file PBI-24-3009-s001.doc]

**Supplement:**

**Figure S1 – qRT-PCR analysis of A. thaliana wild-type and transgenic lines.** Expression data was normalized to the reference AtActin2 (AtACT). The heat map shows mean relative expression levels of the indicated genes of interest (GOIs) compared to AtACT. Expression levels are color-coded from gray to green. Arrows indicate whether the fold-change in expression relative to wild type (Col-0) is higher (↑) or lower (↓) than the natural variability observed in wild type. A dash (−) indicates a fold-change within the range of relative deviation in wild type.
gene abbreviations: . PAL = phenylalanine ammonia-lyase; C4H = cinnamic acid 4-hydroxylase; 4CL = 4-coumarate-CoA ligase; CCoAOMT = caffeoyl CoA 3-O-methyltransferase; C3’H = p-coumaroyl shikimate 3’-hydroxylase; HCT = hydroxycinnamoyl-coenzyme A shikimate:quinate hydroxycinnamoyl-transferase.

**Table S1**. Used oligonucleotides for qRT-PCR analysis.

| **Primer name** | **Primer sequence (5‘-3‘)** | **Gene** |
| --- | --- | --- |
| AtPAL1_RT_F | AACGGAGGAGGAGTGGACG | At2g37040 |
| AtPAL1_RT_R | CTTTCATTTGCTCCGCTGC |  |
| AtACT2_RT_F | GGTAACATTGTGCTCAGTGGTGG | AT3g18780 |
| AtACT2_RT_R | GGTGCAACGACCTTAATCTTCAT |  |
| AtC4H_RT_F | ATTGCGAGTTCTAAGCCTAC | At2g30490 |
| AtC4H_RT_R | TCGTTGATTTCTCCCTTCTG |  |
| At4CL1_RT_F | ACCCAACCAAGGAAACAAAC | At1g51680 |
| At4CL1_RT_R | CTTCATCACAAAGGCTCATTAC |  |
| AtHCT_RT_F | CAAGGAGGATGGGAACACTGTC | At5g48930 |
| AtHCT_RT_R | CCTTTCCCACTGATCTCCACAC |  |
| AtC3'H_RT_F | TATGAACGCTGAAGGTGTTG | At2g40890 |
| AtC3'H_RT_R | CGGGATGTGTTCAGCTATTG |  |
| CCoAOMT1_RT_F | ACCACAAGCGTTTGATCG | At4g34050 |
| CCoAOMT1_RT_R | CTTCCTCATTGGTGCATCAG |  |
| AtMYB72_RT_F | TTGCTGGATTGTTGAGATGTG | At1g56160 |
| AtMYB72_RT_R | AGGGTTTGATGGTAGTGAATG |  |
| AtF6H1_RT_F | CTCAGCCTCTTCTTTGTCTC | At3g13610 |
| AtF6H1_RT_R | AAGCCTCCTCACCATCTTC |  |

**Material and Methods**

**Plant cultivation**

*Arabidopsis thaliana* Col-0 wild-type and transgenic seeds were sown on VM-type soil (Einheitserde Werksverband), transplanted after 2 weeks into new pots and cultivated under short day conditions (8 h light and 16 h darkness at 22 °C) for six weeks before further analysis. *Nicotiana benthamiana* (wild type and OE-line) seeds were grown on ED73-type soil (Einheitserde Werksverband) under long day conditions (16 h light and 8 h darkness at 22 °C) for 5–6 weeks prior to *Agrobacterium*-mediated transformation.

**Real-time quantitative reverse transcription PCR (qRT-PCR)**

RNA from frozen and ground plant material was extracted as previously described by Piotr Chomczynski & Nicoletta Sacchi (1987). cDNA was reverse transcribed as recommended by the manufacturer using 9-mer random oligonucleotides. qPCR was performed as previously described by*.* Beyer et al.(2019). The used primers are listed in table S1.

**Scopoletin and scopolin extraction**

To extract scopoletin and scopolin, plant material was frozen in liquid nitrogen, ground and weighed. 200 mg of plant material was extracted with 1 mL of 90 % MeOH overnight. 800 µL of the supernatant was evaporated using an Eppendorf Concentrator plus and reconstituted in 100 µL of 100 % MeOH.

**High performance liquid chromatography (HPLC) measurement**

HPLC analysis of *A. thaliana* samples was performed as previously described by Beesley et al. (2023).

**Transient transformation of *N. benthamiana***

*Agrobacterium*-mediated transient transformation of *N. benthamiana* leaves was performed as described by Beesley et al. (2023). In short, *A. tumefaciens* (AGL01) harboring the silencing suppressor p19 (Voinnet et al., 2003) or the gene of interest were grown to an OD_600_ of 1 or 0.5, respectively. Prior to infiltration in *N. benthamiana* leaves, p19-Agrobacteria were mixed with the bacteria carrying the gene of interest in equal proportions, e.g., for three constructs it was p19:AtMYB72:AtF6H1 (1:1:1). Three days after infiltration, leaves were harvested for further analysis.

**Stable transformation of *N. benthamiana***

For the stable transformation of *N. benthamiana*, the coding sequence of AtF6’H1 was cloned into the DNA vector pSITE-4NB (Chakrabarty et al., 2007) providing an N-terminal mRFP-tag. *Agrobacterium*-mediated stable transformation of *N. benthamiana* plants was performed as described for *N. tabacum* by Fisher and Guiltinan (1995) with slight modifications. Fully developed leaves from four-week-old *N. benthamiana* plants were sterilized in 70 % EtOH for 30 s and then in bleach (7.5 %) for 20 min, followed by five rinses with ddH_2_O. Leaves were cut into pieces of ~1 cm² and incubated in a Agrobacteria (AGL01; OD_600_ = 0.8) suspension for 30 min. After washing with MS medium (4.4 g/L MS salts with vitamins (Duchefa), 30 g/L sucrose, 1 mg/L 6-benzylaminopurine, 0.1 mg/L 1-naphthylacetic acid, pH 5.6–5.8), leaf discs were dried on sterile filter paper and placed on co-cultivation plates for seven days. Following co-cultivation, leaf discs were transferred to selection medium (4.4 g/L MS salts with vitamins (Duchefa), 30 g/L sucrose, 400 mg/L kanamycin, 400 mg/L cefotaxime, pH 5.6–5.8) until shoot apical meristems emerged (2-3 cm long). Shoots were cut and transferred to rooting medium (2.2 g/L MS salts with vitamins (Duchefa), 5 g/L sucrose, 400 mg/L kanamycin, pH 5.6–5.8) to initiate root growth. After roots reached about 4-5 cm in length, the plants (explants) were transplanted into Einheitserde® and regularly watered and fertilized until seed set.

**Stable transformation of *A. thaliana***

The *AtF6’H1* overexpressing line was previously generated (Beesley et al., 2023). For stable transformation of *A. thaliana* lines overexpressing *AtMYB72* or co-overexpressing *AtMYB72* and *AtF6H1* the floral dip method was used (Clough and Bent, 1998). For single overexpression of AtMYB72 (At1g56160) the DNA vector was generated using Gateway Cloning® as described by the manufacturer (Thermo Fisher). The pB7WG2D vector (Karimi et al., 2002) was used as a backbone. For cloning of co-overexpressing lines, Gibson Assembly (Gibson et al., 2009) was used. The pK7GWIWG vector (Karimi et al., 2002) was used as backbone and cut with restriction enzymes (Thermo Fisher) *XhoI* and *KpnI* according to the manufacturer. *AtMYB72* was cloned from previously generated single expression vector (pB7WG2D *AtMYB72*) together with the viral 35S-promotor so that pK7GWIWG was modified for overexpression. *AtF6’H1* was cloned from the previously described pSite *RFP*-*AtF6’H1* vector.

**BY-2 feeding**

*AtF6’H1overexpressing Nicotiana tabacum* BY-2 suspension cells were generated previously (Beesley et al., 2023). Suspension cells of this line were cultivated as described by Beesley et al (2023). In short, 2 mL of cell culture were weekly transferred into 30 mL fresh modified MS medium (4.3 mg/L MS basal salts (Duchefa), 30 g/L Sucrose, 0.2 g/L KH2PO4, 0.1 g/L myo-inositol, 200 μg/L 2,4-D, 1 mg/L thiamine/HCl) under sterile conditions. For feeding experiments, one-week-old cells were set to a packed cell volume of 30 %. Then, 8 mL of the cells were transferred to 100 mL of fresh medium. The cell suspension was incubated in the dark at 26 °C and shaking at 120 rpm. Nine days after the transfer, the cell cultures were fed with 1 mM ferulate in 0.2 % EtOH or 0.2 % EtOH as control. At indicated timepoints, cells were harvested by vacuum-filtration, ground in liquid nitrogen and extracted with 90 % (v/v) MeOH.

**Detailed Figure Legend**

**Figure 1. AtMBY72 overexpression increases scopoletin and scopolin content in planta**. (a) Simplified scheme of scopoletin biosynthesis in Arabidopsis. Green highlights steps enhanced by AtMYB72. Enzymes: PAL = phenylalanine ammonia-lyase; C4H = cinnamic acid 4-hydroxylase; 4CL = 4-coumarate-CoA ligase; CCoAOMT = caffeoyl CoA 3-O-methyltransferase; C3’H = p-coumaroyl shikimate 3’-hydroxylase; HCT = hydroxycinnamoyl-coenzyme A shikimate:quinate hydroxycinnamoyl-transferase; CCR = cinnamoyl-CoA reductase; **F6’H1 = feruloyl-CoA 6’-hydroxylase**; COSY = coumarin synthase; UGT = UDP-glycosyltransferase; BGLU = ß-glycosidase. (b) Transgenic BY2-tabacco suspension cells overexpressing *AtF6’H1* were fed with 1 mM ferulate or 0.2 % EtOH (control). Cells were harvested at indicated timepoints. Scopoletin (left, light blue) and scopolin (right, dark blue) content was analyzed via HPLC. (d, f) Images of transient transformed *N. benthamiana* wild-type leaves (c) and rosettes of 6-week-old *A. thaliana* (e) wild-type and transgenic lines overexpressing *AtMYB72*, *AtF6’H1* or co-overexpressing both genes (*MYB72/F6’H1*-OE) were taken under UV-light. (d, f) HPLC analysis of methanolic extracts from *N. benthamiana* (d) and *A. thaliana* (f) leaves as in (c, e), quantifying scopoletin (light blue) and scopolin (dark blue). (g) Relative expression of *AtMYB72* and *AtF6’H1* in leaves of 6-week-old *A. thaliana* plants (wildtype and transgenic lines, as indicated). Transcript levels were normalized to *AtActin2* (*AtACT*). Error bars in panels (b, d, f, g) represent the standard deviation of at least three independent biological replicates.

References

Beesley, A., Beyer, S. F., Wanders, V., Levecque, S., Bredenbruch, S., Habash, S. S., Schleker, A. S. S., Gätgens, J., Oldiges, M., Schultheiss, H., Conrath, U. and Langenbach, C. J. G. (2023) Engineered coumarin accumulation reduces mycotoxin-induced oxidative stress and disease susceptibility. *Plant biotechnology journal* **21**, 2490–2506.

Beyer, S. F., Beesley, A., Rohmann, P. F. W., Schultheiss, H., Conrath, U. and Langenbach, C. J. G. (2019) The Arabidopsis non-host defence-associated coumarin scopoletin protects soybean from Asian soybean rust. *The Plant journal for cell and molecular biology* **99**, 397–413.

Chakrabarty, R., Banerjee, R., Chung, S.-M., Farman, M., Citovsky, V., Hogenhout, S. A., Tzfira, T. and Goodin, M. (2007) PSITE vectors for stable integration or transient expression of autofluorescent protein fusions in plants: probing Nicotiana benthamiana-virus interactions. *Molecular plant-microbe interactions MPMI* **20**, 740–750.

Clough, S. J. and Bent, A. F. (1998) Floral dip: a simplified method for Agrobacterium-mediated transformation of Arabidopsis thaliana. *The Plant journal for cell and molecular biology* **16**, 735–743.

Fisher, D. K. and Guiltinan, M. J. (1995) Rapid, efficient production of homozygous transgenic tobacco plants withagrobacterium tumefaciens: A seed-to-seed protocol. *Plant Mol Biol Rep* **13**, 278–289.

Gibson, D. G., Young, L., Chuang, R.-Y., Venter, J. C., Hutchison, C. A. and Smith, H. O. (2009) Enzymatic assembly of DNA molecules up to several hundred kilobases. *Nature methods* **6**, 343–345.

Karimi, M., Inzé, D. and Depicker, A. (2002) GATEWAY vectors for Agrobacterium-mediated plant transformation. *Trends in plant science* **7**, 193–195.

Piotr Chomczynski & Nicoletta Sacchi Single-step method of RNA isolation by acid guanidinium thiocyanate-phenol-chloroform extraction.

Voinnet, O., Rivas, S., Mestre, P. and Baulcombe, D. (2003) An enhanced transient expression system in plants based on suppression of gene silencing by the p19 protein of tomato bushy stunt virus. *The Plant journal for cell and molecular biology* **33**, 949–956.
